# Heterogeneity of PD-L1 expression in non-small cell lung cancer: Implications for specimen sampling in predicting treatment response

**DOI:** 10.1016/j.lungcan.2019.06.005

**Published:** 2019-08

**Authors:** Alexander Haragan, John K. Field, Michael P.A. Davies, Carles Escriu, Aaron Gruver, John R. Gosney

**Affiliations:** a Department of Molecular and Clinical Cancer Medicine, University of Liverpool, William Henry Duncan Building, 6 West Derby Street, Liverpool, L7 8TX, UK; b The Clatterbridge Cancer Centre, Bebington, Wirral, CH63 4JY, UK; c Eli Lilly and Company Corporate Center, 893 Delaware St, Indianapolis, IN, 46225, USA; d Department of Cellular Pathology, Royal Liverpool University Hospital, Duncan Building, Daulby Street, Liverpool, L7 8XP, UK

**Keywords:** PD-L1, Programmed-death ligand-1, NSCLC, Heterogeneity, Nodal metastases

## Abstract

**Objectives:**

PD-L1 expression on tumour cells can guide the use of anti-PD-1/PD-L1 immune modulators to treat patients with non-small cell lung cancer (NSCLC). Heterogeneity of PD-L1 expression both within and between tumour sites is a well-documented phenomenon that compromises its predictive power. Our aim was to better characterise the pattern and extent of PD-L1 heterogeneity with a view to optimising tumour sampling and improve its accuracy as a biomarker.

**Materials and methods:**

Expression of PD-L1 was assessed by immunochemistry using the SP263 clone in 107 resected primary NSCLCs and their nodal metastases. Intra-tumoural heterogeneity, defined as ‘small-scale’ (mm²), ‘medium-scale’ (cm²) and ‘large-scale’ (between tumour blocks), was assessed by digital imaging using a novel ‘squares method’. Inter-tumoural heterogeneity between the primary tumours and their nodal metastases and between N1 and N2 nodal stages was also assessed.

**Results:**

The majority of tumours demonstrated intra-tumoural heterogeneity (small-scale 78%, medium-scale 50%, large-scale 46%). Inter-tumoural heterogeneity between the primary and nodal metastases was present in 53% of cases and, in 17%, between N1 and N2 disease. These differences were occasionally sufficient to lead to discrepancy across the ≥1%, ≥25% and ≥50% cut-offs used to guide therapy.

**Conclusion:**

Heterogeneity of PD-L1 expression is common, variable in scale and extent, and carries significant implications for its accuracy as a predictive biomarker. Extensive sampling reduces, but cannot eliminate, this inaccuracy.

## Highlights

• PD-L1 expression was assessed for heterogeneity in 107 NSCLC patients.
• Intra-tumoural heterogeneity was observed in 78% of cases.
• Inter-tumoural heterogeneity was observed in 53% of cases.
• 23% of cases had clinically relevant changes between primary and secondary tumours.
• Sample site selection is an important consideration for testing PD-L1.

## 1 Introduction

The treatment of patients with non-small cell lung cancer (NSCLC)^1^ has been revolutionised by the emergence of immune-checkpoint inhibitors or ‘immune modulators’ (IMs), particularly those targeted against tumours exploiting the PD-1/PD-L1 (programmed death-1; programme death ligand-1) checkpoint as a mechanism of immune escape. [[1], [2], [3], [4]] Currently, the level of expression of PD-L1 as detected by immunohistochemistry (IHC) is the only accepted biomarker for guiding the use of IMs to treat NSCLC, numerous clinical trials having shown that expression of PD-L1 by the tumour or tumour-associated immune cells is related to response to the drug [[1], [2], [3], [4], [5], [6]]. Despite its rapid implementation in the routine profiling of NSCLC, PD-L1 expression as a predictor of response has several weaknesses compromising its predictive power. Amongst these are the multiplicity of assays, differing expression level percentage cut-offs for assigning ‘positive’ status and guiding therapy, and the biological fact that PD-L1 expression is heterogeneous [7,8]. These drawbacks have resulted in a confusing, mixed status of PD-L1 IHC as both a companion and complementary diagnostic and have raised justifiable doubts about its efficacy [[7], [8], [9], [10], [11], [12], [13]]. Despite these doubts, reliance on PD-L1 IHC for predicting response of NSCLC to IMs means it is imperative that, in the absence of alternative proven biomarkers, every effort should be made to maximise its utility in guiding clinical decision-making.

Crucial to addressing the problem of heterogeneity in the context of assessing PD-L1 expression is knowing how best to sample the tumour. Many clinical specimens used for the diagnosis, classification and profiling of NSCLC, including endoscopic bronchial ultrasound (EBUS)-guided aspirates, endobronchial and transthoracic needle biopsies, are very small, and sampling error is problematic in obtaining maximum accuracy. [8,[14], [15], [16]] Understanding the pattern and extent of heterogeneity of PD-L1 expression is a prerequisite for developing and adapting approaches to tumour sampling and ultimately increasing the predictive power of the test. In order to help address this challenge, we thought it would be of value to try and assess the pattern and extent of intra-tumoural and inter-tumoural heterogeneity of PD-L1 expression and thereby develop some practical guidance for those obtaining these crucial specimens.

## 2 Materials and methods

### 2.1 Specimens studied

We studied 107 resected NSCLCs consecutively collected and archived by the Liverpool Lung Project (LLP) between 2009 and 2014. Tumours were classified according to the then current World Health Organisation 2015 criteria [17] and staged according to the then current seventh edition of the Union for International Cancer Control (UICC) TNM staging system [18]. Every primary pulmonary tumour was accompanied by metastases in lymph nodes at stations 10–14 (N1), 1–9 (N2) or both. Between one and two blocks of formalin-fixed, paraffin wax-embedded (FFPE) tissue were studied from the primary tumour and between one and five blocks from all involved lymph nodes. Accompanying clinical data were available within the LLP database, from case-note review. Details of these 107 tumours are given in Table 1. Ethical approval was granted by the Liverpool Research Ethics Committee (reference number 97/141).

**Table 1:** Demographic and clinical characteristics of patients and pathology of tumours.

Clinical Characteristics	N (%)
**Total number**	107 (100)
**Morphology**	
ADC	63 (59)
SCC	44 (41)
**Median Age (at diagnosis)**	68 (Range 46-84)
**Gender**	
Male	66 (62)
Female	41 (38)
**Smoking Status**	
Never	11 (10)
Light (≤20 CSMPYs)	11 (10)
Heavy (>20 CSMPYs)	85 (80)
Stage*(at diagnosis)	
T1**	34 (32)
T2***	54 (50)
T3	15 (14)
T4	4 (4)
**Morphology subtype**	
ADC – Acinar	31 (29)
ADC – Mucinous	16 (15)
ADC – Solid	14 (13)
ADC – Papillary	2 (2)
SCC - poorly differentiated	6 (5)
SCC - moderately differentiated	38 (36)
Nodal Stage*(at diagnosis)	
N1	63 (59)
N2	44 (41)
N1 & N2	33 (31)

* Staged according to TNM 7^th^ edition.¹⁸ ** Includes T1a-T1c, *** Includes T2a + T2b. ADC, adenocarcinoma; SCC, squamous cell carcinoma; CSMPYs, combined smoking pack years.

### 2.2 Detection and assessment of PD-L1 expression

Serial sections 4 μm thick were stained with haematoxylin and eosin (H&E) for assessment of general morphology and immuno-stained for PD-L1 using the Ventana SP263 antibody clone with a validated kit and protocol. [19] Slides were scanned at x20 magnification to create digital images using the Aperio CS2 Scanscope slide scanner and Aperio Scancope console software [20]. Images were viewed using either Aperio ImageScope or the opensource QuPath software package [20,21].

Expression of PD-L1 was assessed according to the Roche Ventana SP263 interpretation guide [22] by two pathologists trained and experienced in its interpretation and a concordant score agreed in all cases. The number of PD-L1+ve tumour cells as a proportion of the total number of tumour cells (the *tumour proportion score, TPS*) was expressed as a percentage.

### 2.3 Assessment of heterogeneity

Intra-tumoural heterogeneity was quantified comparing (a) different samples from the same tumour and (b) different samples from its nodal metastases. Inter-tumoural heterogeneity was assessed comparing samples from the primary tumour with samples from its nodal metastases, and samples from separate nodal metastases.

#### 2.3.1 Intra-tumoural heterogeneity

**Fig. 1:**
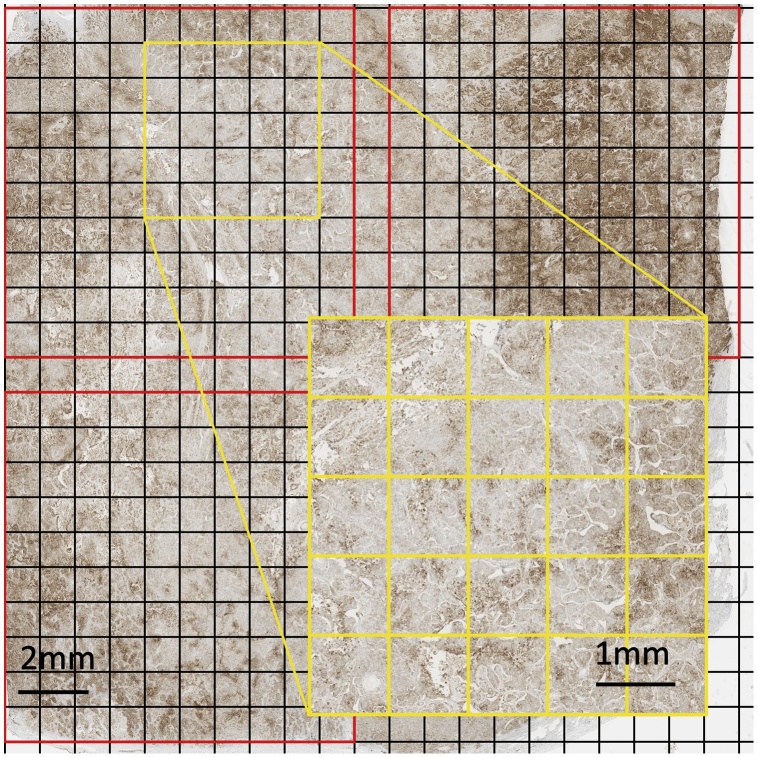
Title: Intra-tumoural heterogeneity of PD-L1 as assessed by the ‘squares method’. Description: A section of primary NSCLC immunolabelled for PD-L1 (SP263) overlaid with non-overlapping, 1 cm² grids (outlined in red), each divided into 100 1 mm squares. The yellow inset highlights a group of 20 1 mm squares. Every 1 mm square was individually assessed for PD-L1 expression and constituted a different data point for examining heterogeneity. NSCLC, non-small cell lung cancer; PD-L1, programmed death ligand 1. (For interpretation of the references to colour in this figure legend, the reader is referred to the web version of this article).

**Fig. 2:**
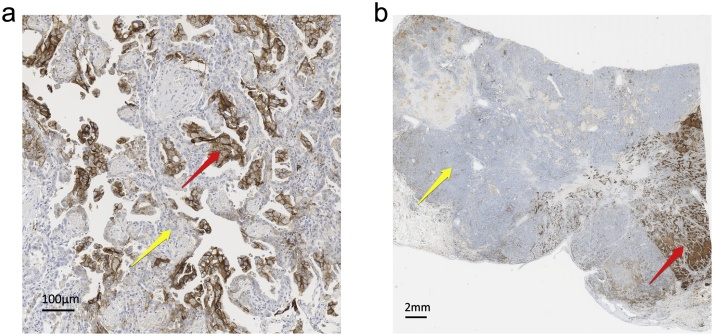
**(a, b)**Title: Intra-tumoural heterogeneity of PD-L1 expression; primary tumour. Description: Two sections of a primary NSCLC immunolabelled for PD-L1 (SP263). Fig. 2a demonstrates small scale heterogeneity. Fig. 2b demonstrates large scale heterogeneity. The red arrows highlight tumour cells strongly positive for PD-L1, the yellow arrows highlight tumour cells showing no expression. NSCLC, non-small cell lung cancer; PD-L1, programmed death ligand 1. (For interpretation of the references to colour in this figure legend, the reader is referred to the web version of this article).

First, small scale heterogeneity, defined as heterogeneity within an approximately 1 cm² area of tumour was assessed using a grid split into 1 mm squares that was overlaid on to the section (Fig. 1). Only sections containing a continuous area of viable tumour were assessed; zones of confluent necrosis or fibrosis were avoided and sections in which these were extensive were not used. The PD-L1 TPS was assessed for every 1 mm square to give 100 readings for each area of 1 cm². Between one and three 1 cm squares were assessed in every section studied by this ‘squares method’ for primary tumours. Second, medium scale heterogeneity, defined as heterogeneity between 1 cm squares, was examined for primary tumours to give a broader assessment of intra-tumoural heterogeneity. Finally, large scale heterogeneity, defined as heterogeneity between different tissue blocks, was assessed for primary tumours by scoring the entire viable tumour region within each section. Intra-tumoural heterogeneity of nodal metastases was assessed by scoring any overlaid 1 mm square with ≥100 tumour cells. Illustrative examples of intra-tumoural heterogeneity are shown in Fig. 2.

#### 2.3.2 Inter-tumoural heterogeneity

With the above data collected for primary and metastatic tumours individually, inter-tumoural heterogeneity, that is variability between primary tumours and their nodal metastases as well as between different nodal metastases, could then be assessed. For both primary and secondary tumours, PD-L1 TPS for inter-tumoural comparison was calculated from all available PD-L1 scored tissue.

Finally, an average PD-L1 TPS was calculated for all tumours studied and these were then stratified according to the ≥1%, ≥25%, ≥50% cut-offs used to guide prescription of IMs. [1,3,[5], [6], [7]]

### 2.4 Statistical analysis

Statistical analysis was performed using IBM SPSS statistics software, version 25 (IBM Corp). Variation of data was described using index of dispersion (IOD) and compared using co-efficient of variation (COV). Comparison of COV was performed according to Forkman. [23] All significances were taken as p<0.05.

## 3 Results

### 3.1 Study population

Basic demographic, clinical and pathological details of the 107 subjects and tumours studied are given in Table 1. No patient from whom these tumours were resected had received neoadjuvant chemotherapy or radiotherapy treatment.

### 3.2 Intra-tumoural heterogeneity within primary tumours

#### 3.2.1 Small and medium scale heterogeneity

There was sufficient quantity and quality (>1 cm² of continuous viable tumour cells) for assessment by the ‘squares method’ in 50 of the primary tumours and in 19 of these there was sufficient tissue for 2 blocks to be studied. In 16 tumours, there was sufficient tissue (>2cm² and ≥200 viable tumour cells) for assessment of multiple, non-overlapping 1 cm² squares in a single section (two squares in 14 and three squares in 2) such that 87 1 cm² squares were ultimately examined. [Dataset]

Data on small scale heterogeneity, within an area of 1 cm², are summarised in Table 2. In 68 primary tumours (78%) the IOD was >1. In the 66 primary tumours scoring a TPS of ≥1%, 32 (48%) had a standard deviation (SD) greater than their mean.

**Table 2:** Intra-tumoural heterogeneity of PD-L1 expression: Large scale comparing PD-L1 TPS from two matched whole sections of primary NSCLC. Small scale PD-L1 TPS variability in primary NSCLC and metastatic deposits in regional lymph nodes.

Large Scale		Small Scale		
Change of TPS	Number of cases (%)		Primary N (%)	Metastatic N (%)
No (0%)	33 (54)	SD>Mean*	32 (48)	6 (23)
Yes (≥1%)	28 (46)	**IOD >1**	68 (78)	19 (73)
>10%	10 (16)	**IOD = 1**	0 (0)	1 (4)
≤10%	18 (30)	**IOD <1**	19 (22)	6 (23)
Range	4-30	**COV (average)**	146	98
**Clinical Group Change**	**Number of cases (%)**			
No	56 (92)			
Yes	5 (8)			
≥1%	0 (0)			
≥25%	1 (2)			
≥50%	4 (7)			

* For cases with a TPS ≥ 1%. PD-L1, programmed death ligand 1; NSCLC, non-small cell lung cancer, SD, standard deviation; IOD, index of dispersion; COV, coefficient of variation; TPS, tumour proportion score.

Of the 16 specimens assessed for medium scale heterogeneity, 7 specimens (44%) had no change in PD-L1 TPS, 9 specimens (56%) had a PD-L1 TPS change of ≥1% including 3 specimens (19%) with a PD-L1 TPS change of ≥10%. For 2 specimens (12.5%), the difference was sufficient to move the PD-L1 TPS across the ≥50% clinical guidance cut-off.

#### 3.2.2 Large scale heterogeneity

There was sufficient tissue in 61 primary tumours to permit assessment of large scale heterogeneity, that is variability between two tissue blocks. In 33 of these (54%), there was no difference in TPS between the two blocks. 28 cases (46%) had a TPS change of ≥1% and 17 cases (28%) had a TPS change of ≥10%. For 5 cases (8%), the difference was sufficient to move the PD-L1 TPS across a clinical guidance cut-off, (1 across ≥25%, 4 across ≥50%). These data are summarised in Table 2.

### 3.3 Intratumoural heterogeneity within nodal metastases

In the nodal metastases from 26 cases there was sufficient assessable tumour tissue (≥100 viable tumour cells) for assessment of heterogeneity by the ‘squares method’. In 19 metastases (73%), the IOD was >1. In the 23 nodal metastases scoring a PD-L1 TPS of ≥1%, 6 (23%) had a SD greater than their mean. These results are summarized in Table 2.

Intra-tumoural heterogeneity within primary tumours as assessed by the ‘squares method’ had a greater COV than it did in their nodal metastases, but the difference was not statistically significant (146 vs 98; p = 0.3706).

### 3.4 Inter-tumoural heterogeneity

#### 3.4.1 Primary versus matched nodal metastases

**Fig. 3:**
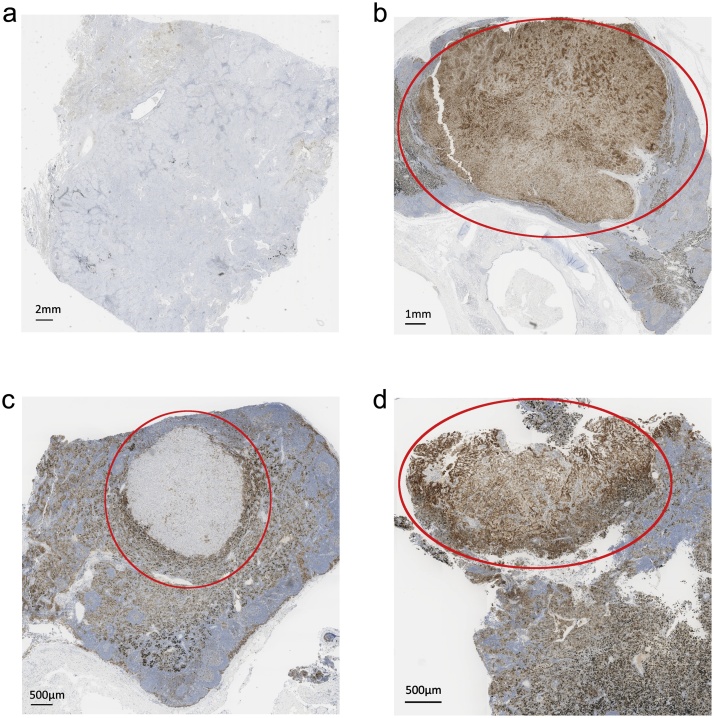
**(a–d)**Title: Inter-tumoural heterogeneity of PD-L1 expression. Description: Sections of a primary NSCLC and nodal metastases immunolabelled for PD-L1 (SP263). Fig. 3a demonstrates no expression (0% TPS) by the primary tumour, Fig. 3b demonstrates diffuse expression (100% TPS) in a nodal metastasis from the same patient as indicated by the red oval. Fig. 3c demonstrates minimal expression (<1% TPS) of PD-L1, as indicated by the red oval, in a metastasis in an N1 lymph node, Fig. 3d demonstrates expression by almost all of the cells (near 100% TPS) as exemplified by the zone in the red oval, in a metastasis in a different (N2) lymph node from the same patient. NSCLC, non-small cell lung cancer; PD-L1, programmed death ligand 1; TPS, tumour proportion score. (For interpretation of the references to colour in this figure legend, the reader is referred to the web version of this article).

PD-L1 expression by the primary tumour and its nodal metastases was compared in all 107 tumours studied. In 50 tumours, there was no difference. In the remaining 57 (53%) there was a difference of ≥1%, with 30 displaying higher expression by the primary than by their nodal metastases and 27 the converse. The median difference in TPS between the primaries and their nodal metastases was 10% (range 1–94). In 25 cases (23%), this difference was sufficient to move the TPS across a clinical guidance cut-off. In 13 cases (12%), the PD-L1 TPS was ≥1% in the primary but 0% in its metastases. In 3 cases (3%), the PD-L1 TPS was 0% in the primary, but ≥1% in its metastases. These data are summarised in Table 3 and example shown in Fig. 3a and 3b.

**Table 3:** Inter-tumoural PD-L1 heterogeneity: Comparison of PD-L1 TPS between primary NSCLC and matched nodal metastases, and comparison of PD-L1 TPS between matched nodal metastatic deposits.

Primary vs. metastatic tumour PD-L1 TPS				N1 vs N2 metastases PD-L1 TPS			
TPS Change N (%)		Clinical Change N (%)		TPS Change N (%)		Clinical Change N (%)	
**0%**	50 (47)	**No**	82 (77)	**0%**	29 (83)	**No**	25 (81)
**≤10%**	31 (29)	**Yes**	25 (23)	**≤10%**	3 (8.5)	**Yes**	6 (19
**>10%**	26 (24)	**≥1%**	12 (11)	**>10%**	3 (8.5)	**≥1%**	3 (10)
**Total**	57 (53)	**≥25%**	3 (3)	**Total**	6 (17)	**≥25%**	0 (0)
**Range**	94 (1-95)	**≥50%**	10 (9)	**Range**	90 (5-95)	**≥50%**	3 (10)

PD-L1, Programmed death ligand 1; TPS, tumour proportion score; NSCLC, non-small cell lung cancer.

#### 3.4.2 Variation between nodal metastases

In 35 of the tumours studied, there was sufficient tissue from nodal metastases for variation in PD-L1 expression between them to be studied; N1 vs N1 in four cases and N1 vs N2 in 31. In 29 cases (83%), there was no difference between stations, including N1 vs N1. In the remaining 6 cases (17%), the difference between N1 and N2 stations was ≥10%. In all of these, it was sufficient to move the TPS across a cut-off. These results are summarised in Table 3 and example shown in Fig. 3c and 3d.

## 4 Discussion

The extent of expression of PD-L1 as detected by IHC is currently the only clinically-validated means of determining the likely response of NSCLC to IMs. [[1], [2], [3], [4]] Characterising and understanding the strengths and limitations of PD-L1 expression in this context are crucial to improving its predictive power.

Several studies have attempted to quantify how many biopsy specimens of a NSCLC are required to provide accurate coverage of PD-L1 expression within a tumour [[24], [25], [26]], many concluding rather obviously that, for example, multiple core biopsies are likely to provide greater accuracy than one or two and that tumours displaying marked heterogeneity still present significant difficulty. The present study concurs with this; increasing quantities of tissue for assessment will clearly improve its accuracy, but even a whole tissue section might still not be representative of the entire tumour. Even the detailed and extensive study of a large series of tumours that we describe here fails to reveal any particular pattern to this heterogeneity, which seems highly variable in extent and scale. This observation holds for not only the primary tumour, but also its nodal metastases. Intra-tumoural heterogeneity is unlikely to be random, but reflects ill-understood aspects of the interaction between the tumour and the immune environment and underlying clonal variation within the tumour. More sophisticated analytical approaches are required to untangle these relationships.

Inter-tumoural heterogeneity of PD-L1 expression is a no less significant challenge in terms of achieving high accuracy and predictive power. Several studies have examined PD-L1 expression between a primary NSCLC and its metastases [[27], [28], [29]] and, though approaches and methodologies differ, the general consensus of these is that expression of PD-L1 varies between tumour sites in the majority of cases. Our investigation supports this, revealing a fairly equal divide between tumours in which expression of PD-L1 ‘increases’ or ‘decreases’ as they metastasise into regional lymph nodes, with complete loss of PD-L1 expression during metastasis occurring with more frequency than its apparent *de novo* expression in the environment of the node. An important observation is that this variation between the primary and its metastases was often sufficient to cross one of the cut-off thresholds used for guiding management. This raises the important question of which score should be acted upon. It would seem reasonable to assume that a tumour deposit expressing high levels of PD-L1 would be likely to respond to an IM, whereas a different deposit expressing low levels would not; this might be one cause for variable response of different lesions of a disseminated tumour. On the grounds that any response would be beneficial, whenever such variability is apparent, it would seem appropriate to act on the highest score.

Ultimately, in the context of NSCLC, expression of PD-L1 is being determined in an already heterogeneous population of tumour cells further affected by their interaction with the tumour micro-environment (TME) [30]. Immune escape of NSCLC is thought to require, in addition to PD-L1 expression, specific conditions within the TME, such as the proximity of CD8+ cytotoxic T-cell lymphocytes and a non-suppressive immune environment [[31], [32], [33], [34]]. With this in mind, it is not surprising that PD-L1 expression varies between a primary NSCLC and its nodal metastases; the environment in the lung, especially the immune environment, is very different from that in a lymph node.

Irrespective of its nature, bronchoscopic, transthoracic needle or EBUS-guided, there is a high risk that a single diagnostic sample of a NSCLC, primary or metastatic, will be inadequately representative for determining something as heterogeneous as PD-L1 expression. Notwithstanding the obvious conclusion that greater accuracy is more likely with a larger specimen and, ideally, multiple biopsies or aspirates from multiple points within a tumour, it is difficult to see how this challenge can be easily overcome. Not surprisingly, therefore, efforts are being made to find an alternative or, more likely, complementary biomarkers to use in conjunction with PD-L1 expression and improve predictive capabilities, with much current interest focussed on tumour mutational burden (TMB) or assessment of the immune environment of the tumour. [[35], [36], [37], [38]]

In the interim, however, with PD-L1 expression still the only validated biomarker for predicting response of NSCLC to anti-PD-1/PD-L1 IMs, an optimal approach to improved tumour sampling may be guided by the intended therapeutic target. Neoadjuvant treatment of NSCLC by IMs is being assessed in current clinical trials [39] and extensive sampling of primary tumour in this setting would seem prudent. Metastasis, however, is a reflection of evolution of the tumour, a manifestation of its inherent drive to survival, and it would seem reasonable to assume that the most advanced and potentially successful component of a disseminated tumour would be the most informative in terms of targeting for biopsy [30,40,41]. When metastases are present, therefore, sampling and testing of these in preference to the primary growth, whenever possible, would seem the most scientifically sound approach and most likely to provide informative information.

## Conflict of interests

Dr Alex Haragan: research funded by Eli Lilly and Company via UK North West MRC scheme.

Professor John R Gosney: paid advisor to and speaker for Abbvie, AstraZeneca, Boehringer-Ingelheim, Bristol-Myers Squibb, Diaceutics, Eli Lilly and Company, Merck Sharp & Dohme, Novartis, Pfizer, Roche, Takeda Oncology.

Dr A Gruver is an employee of Eli Lilly and Company.

Prof John K Field: Speaker’s Bureau for AstraZeneca. Advisory Board for Epigenomics, NUCLEIX Ltd., AstraZeneca and iDNA. Grant Support from Janssen Research & Development and LLC.

Dr C Escriu and Dr Micheal PA Davies report no conflicts of interest.

## Footnotes

1 Abbreviations: PD-L1, programmed-death-ligand-1; IHC, immunohistochemistry; IM, immuno-modulators; TPS, tumour proportion score; COV, co-efficient of variation; IOD, index of dispersion.
